# Multiparametric‐magnetic resonance imaging (mp‐MRI) of the prostate and Urolift: Identifying artefact size, location and clinical implications

**DOI:** 10.1002/bco2.392

**Published:** 2024-06-01

**Authors:** Cameron James Parkin, Rajeev Jyoti, Peter Chin

**Affiliations:** ^1^ Department of Urology Wollongong Hospital, Illawarra Shoalhaven Local Health District Wollongong NSW Australia; ^2^ Calvary Hospital Universal Medical Imaging Canberra Australia

**Keywords:** artefacts, magnetic resonance imaging, minimally invasive surgical procedures, prostatic hyperplasia, prostatic urethral lift

## Abstract

**Objectives:**

We sought to define the degree of artefact caused by prostatic urethral lift (PUL) on multiparametric‐magnetic resonance imaging (mp‐MRI) to determine the location, size of artefact and if the device could potentially obscure a diagnosis of prostate cancer.

**Methods:**

Ten patients were prospectively enrolled to undergo PUL for treatment of benign prostatic hyperplasia and follow‐up imaging. A standard mp‐MRI protocol using a 3.0 Tesla scanner was performed prior to and following Urolift insertion. Pre‐ and post‐PUL images were compared to measure maximum artefact diameter around each implant in each MRI parameter. A transverse relaxation time weighted (T2) artefact reduction protocol was also evaluated. The location of each artefact was then compared to a separate database of 225 consecutive patients who underwent magnetic resonance guided prostate biopsies.

**Results:**

Artefact occurred around the stainless steel urethral implant component only. Mean T2 artefact maximum diameter was 7.7 mm (sd = 1.71 mm), with an artefact reduction protocol reducing this to 5.4 mm (sd = 1.43). Mean dynamic‐contrast‐enhancement artefact was 10 mm (sd = 2.5 mm), and mean diffusion‐weighted‐imaging artefact was 28.2 mm (sd = 7.8 mm). All artefacts were confined to the posterior transition zone only. In the 225 consecutive patients who had undergone magnetic resonance guided prostate biopsies, there were 55 positive biopsies with prostate cancer, with 13 cases found in the transition zones and no cancer identified solely in the posterior transitional zone.

**Conclusions:**

The stainless steel urethral component of the PUL does cause artefact, which is confined to the posterior transition zone only. PUL artefact occurs in an area of the prostate that has a very low incidence of a single focus of prostate cancer. If there is concern for prostate cancer in the posterior TZ (e.g. if every other area is clear with a high PSA), this area can undergo targeted biopsy.

## 1 INTRODUCTION

Benign prostatic hyperplasia (BPH), alongside prostate cancer, is one of the most common urological conditions treated worldwide.^1^

Traditionally the ‘gold‐standard’ technique in the surgical management of BPH has been a transurethral resection of prostate or TURP. However, whilst its safety and efficacy are well known, it is also known to have several associated side effects including retrograde ejaculation, erectile dysfunction, urethral stricture formation and stress urinary incontinence.^2^, ^3^ This has led to the belief that particularly for young patients who remain sexually active, a TURP may no longer be considered the ‘gold‐standard’ option. As a result, in recent years, there has been a rise in the use of minimally invasive surgical treatment modalities such as the prostatic urethral lift (PUL).^4^

The PUL (UroLift® NeoTract Inc., Pleasanton, CA, USA) is a form of minimally invasive surgery used to treat patients with LUTS secondary to BPH. Its principles are based on widening the prostatic urethra by pinning open the obstructing prostate lobes with nonabsorbable sutures.^5^ It also has the advantage in being able to be performed under local anaesthesia.^6^ The PUL device is comprised of three components: a nonabsorbable suture, a stainless steel urethral tab and a nitinol alloy capsular tab. The device attaches to a rigid cystoscope, which allows the surgeon to deploy the nonabsorbable sutures endoscopically.

With respect to International Prostate Symptom Scores (IPSS), PUL has shown to reduce scores by a mean of 9.6 at 24 months and 7.9 at 60 months as well as improving peak urinary flow rates by 4.2 mL/s.^7^, ^8^, ^9^, ^10^, ^11^ When directly comparing TURP and PUL, the BPH6 study undertook a prospective randomised study of 80 men who underwent either a PUL or TURP for management of BPH.^11^ Whilst TURP was shown to be superior in IPSS and Qmax improvements, PUL resulted in a more rapid recovery, greater preservation of sexual function and a greater overall quality of life improvement.^11^

As the use of PUL continues to grow, so too has the use of MRI in the diagnosis, staging and surveillance of prostate cancer. Multiparametric‐magnetic resonance imaging (mp‐MRI), which incorporates diffusion‐weighted‐imaging (DWI), dynamic‐contrast‐enhancement (DCE) and transverse relaxation time weighted (T2) imaging, has significantly improved the diagnosis of high‐risk prostate cancer and therefore the selection of patients who should undergo a prostate biopsy and eventual definitive treatment.^12^ At time of ultrasound‐guided biopsy, it provides the surgeon with the ability to either cognitively target or utilise computer based software to fuse the suspicious lesions identified on magnetic resonance imaging (MRI) with the live ultrasound findings at time of biopsy.

BPH and prostate cancer often can be intertwined, with meta‐analysis confirming a positive association between BPH and prostate cancer.^13^ As the PUL implant has metallic components, the question has arisen whether artefact generated from the device may obscure the interpretation of MRI for the purpose of diagnosing prostate cancer.^14^ The DWI phase, which plays an important role in the diagnosis of prostate cancer, is particularly known to be prone to metal artefact.^15^

The aim of this study was to determine the degree of artefact that results from the PUL implant and whether this could impede interpretation of clinically significant prostate cancer on MRI.

## 2 MATERIALS AND METHODS

Following ethics committee approval, 10 men due to undergo a PUL procedure for management of BPH were prospectively enrolled in the study. These patients were not suspected of having prostate cancer. To assess the degree of artefact from the PUL, a standard mp‐MRI protocol using a 3.0 Tesla (3 T) scanner was performed prior to PUL and then repeated 1–3 months post device insertion.

These 10 patients then underwent PUL, which were performed by a single high volume surgeon who was involved in Urolift implantation since 2005. The surgeon performs approximately 70 device insertions per year. All implants were inserted anterolaterally into the obstructing lateral lobes of the prostate between the bladder neck and verumontanum until a continuous anterior channel was observed in the prostatic fossa. Typically, this is accomplished with four to six implants.

Pre‐ and post‐PUL images were compared to measure maximum artefact diameter around each implant in T2, DWI and DCE phases. The location and degree of artefact was recorded for each device by a centralised radiologist.

An artefact reduction protocol was used by the centralised radiologist to reduce the size of the artefact created by the metallic PUL implant in the T2 phase. Traditionally, this can be performed through the use of standardised techniques such as lowering the magnet field strength (i.e. using 1.5 T rather than 3 T), using a fast spin‐echo pulse sequence with short echo spacing, increasing the receiver bandwidth, reducing slice thickness and avoiding spectral fat suppression.^16^ In our study, we utilised the multi‐acquisition variable‐resonance image combination selective (MAVRIC‐SL), which is a specialised computer software which aims to do this in a semiautomated process. It is based on a three dimensional fast spin‐echo sequence and a narrow band frequency. The MAVRIC‐SL protocol has previously demonstrated to improve image quality of prostate MRI compared to the conventional MR techniques in reducing signal voids generated from metallic hip joint replacements.^17^

The artefacts recorded from the 10 patients who underwent PUL were then compared to an independent database of 225 consecutive patients who had undergone an MRI and subsequent prostate biopsy for the purpose of investigating a new diagnosis of prostate cancer. These patients underwent cognitive fused biopsies to target suspicious lesions identified on MRI alongside a standard template approach. In those patients who had biopsy‐proven prostate cancer, the locations of diagnosed prostate cancer were then mapped to either the peripheral zone (PZ), transitional zone (TZ) or central zone (CZ). Transitional zone cancer was further categorised to the anterior transitional zone (TZa) or posterior transitional zone (TZp) per the sector mapping outlined in accordance with Prostate Imaging Reporting and Data System (PIRADS version 2.1)^18^

## 3 RESULTS

Ten patients underwent baseline mp‐MRI imaging prior to PUL for the management of BPH. All 10 patients had PIRADS 2 changes only, with no radiological evidence of clinically significant prostate cancer. A mean of 4.9 implants were delivered per patient with a mean prostate volume of 61.9 cc (Table 1). Median follow‐up mp‐MRI was conducted 50.5 days (IQR = 42) post‐procedure. All implants were identified to be positioned within the prostatic urethra and extending outwards through the transition zone and lateral aspect of the peripheral zone of the prostate. There were no cases of implants being positioned incorrectly or outside the transition zone.

**TABLE 1:** Demographics of 10 patients who underwent PUL insertion for the treatment of BPH who underwent multiparametric‐magnetic resonance imaging prior to device insertion.

Number of patients	Mean age (years)	Mean prostate size (cc)	Mean number of PUL implants	Median days between PUL and post‐op MRI
10	63.9	61.9	4.9	50.5

Abbreviations: BPH, benign prostatic hyperplasia; MRI, magnetic resonance imaging; PUL, prostatic urethral lift.

**FIGURE 1:**
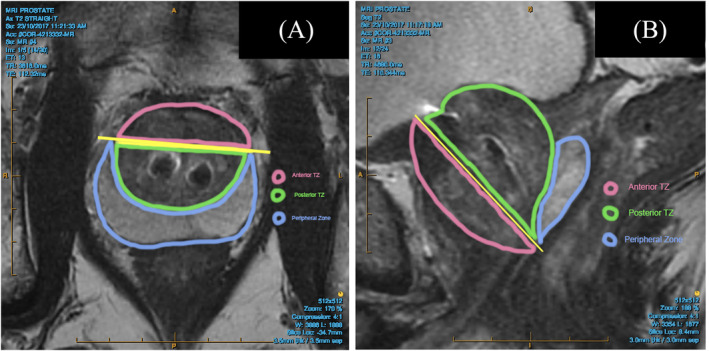
Axial Plane. Horizontal line at the confluence of Peripheral Zone and Transition Zone joining the tips of the Peripheral Zone anteriorly (yellow line). Anterior to the yellow line is the Anterior Transition Zone, posterior to the yellow line is the Posterior Transition Zone. The Peripheral Zone appears much brighter and is easily seen on the T2 phase. Figure 2B ‐ Sagittal Plane midline position. Line from the anterior urethra to the distal extension of the Peripheral Zone (yellow line). Anterior Transition Zone is outlined in pink. Posterior Transition Zone is outlined in green. Peripheral Zone is outlined in blue. [Correction added on 19 June 2024, after first online publication: Figure 1 caption has been corrected.]

**FIGURE 2:**
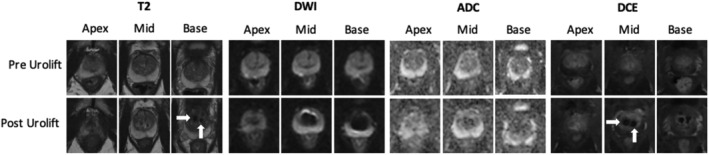
Multiparametic Magnetic Resonance Imaging Pre and Post PUL insertion. Artifact can be appreciated particularly in the T2 phase and DCE phases as dark areas (white arrows). [Correction added on 19 June 2024, after first online publication: Figure 2 caption has been corrected.]

MRI metal artefact was found to occur only around the stainless steel urethral tab of the implant (Figure 1). The nitinol capsular tab caused no visible artefact under any MRI parameter. The urethral component artefact was observed to be confined to the TZp only, as described in Figure 2. The artefact did not affect the imaging of the TZa or PZ in any protocol. The greatest degree of artefact was found in the DWI phase with a mean artefact of 28.17 mm (sd = 7.78; Table 2). The artefact was less in the T2 (mean artefact 7.65 mm, sd = 1.71) and DCE phases (mean artefact 10.04 mm, sd = 2.49). When applying an artefact reduction protocol for the T2 phase, the mean artefact from the device was reduced from 7.65 (sd = 1.71) to 5.35 (sd = 1.43).

**TABLE 2:** Artefact recorded from the prostatic urethral lift device following repeat mp‐MRI.

Variable	Mean (mm)	Median (mm)	sd (mm)	Lower 95% CI for Mean (mm)	Upper 95% CI for mean (mm)
Implant artefact in T2	7.65	7.7	1.71	7.17	8.13
Artefact reduction in T2	5.35	5.05	1.43	4.95	5.75
DWI artefact	28.17	26.5	7.78	22.18	34.15
DCE artefact	10.04	9.40	2.49	9.33	10.74

Abbreviations: DCE, dynamic‐contrast enhancement; DWI, diffuse weighted imaging; mp‐MRI, multiparametric‐magnetic resonance imaging; sd, standard deviation; T2, transverse relaxation time weighted.

After determining the degree and location of artefact generated from the PUL implant, these findings were correlated with a separate database of 225 patients who had undergone MRI and subsequent prostate biopsy for the diagnosis of prostate cancer. The PIRADS findings of this cohort of patients were as follows—PIRADS 2 (*n* = 9, 4%), PIRADS 3 (*n* = 105, 46.7%), PIRADS 4 (*n* = 78, 34.7%), PIRADS 5 (*n* = 33, 14.7%). Fifty‐five of the 225 had positive biopsies containing prostate cancer (Table 3). These were found to be located predominately in the PZ (*n* = 32) and TZ (*n* = 13). Within the transition zone, 10 positive biopsies were in TZa only and none were in TZp only. The single positive biopsy within TZp coincided with a positive sample from the PZ, and, thus, prostate cancer detection would not have been missed in this cohort of patients.

**TABLE 3:** Prostate mapping of 225 patients who underwent MR‐guided biopsies for diagnosis of prostate cancer.

Prostate zone	Gleason 6	Gleason ≥7	Total
PZ	12	20	32
TZ	5	8	13
TZa only	5	5	10
TZp only	0	0	0
TZa + PZ	1	1	2
TZp + PZ	1	0	1
Total	24	31	55

Abbreviations: PZ, peripheral zone; TZ, transition zone; TZa, anterior transition zone; TZb, posterior transition zone; TZp, posterior transitional zone.

## 4 DISCUSSION

Recently, Benedir published images of MRI artefact associated with the PUL implant.^14^ This is important information but is insufficient to determine how urologists should consider this artefact in their often simultaneous management of BPH and prostate cancer. Our results corroborate that only the stainless steel urethral component of the PUL device generates artefact and we further demonstrate that the artefact is located only within the posterior transitional zone of the prostate. The Nitinol capsular tab does not cause artefact in any MRI study phase. There is thus no significant artefact that obscures the anterior transitional zone or peripheral zone, where the majority of prostate cancer occurs.^19^ This is significant as only a very small proportion of prostate cancer has been shown to occur singularly within the TZp, with studies outlining that approximately 70% of prostate cancer occurs in the PZ and 30% in the TZ.^19^, ^20^ This was supported by our series of 255 consecutive MR‐guided prostate biopsies where 23.6% of prostate cancer occurred in the TZ, with no cancer identified solely in the posterior transitional zone. There is also growing evidence to suggest that there is a difference in the clinicopathological behaviour of prostate cancer in the PZ compared to the TZ, with TZ cancers appearing to behave in a less aggressive fashion.^21^, ^22^

The dynamic nature of mp‐MRI and use of artefact reduction protocols provides clinicians with additional tools to aid in the diagnosis of prostate cancer which may be obscured by artefact from devices such as the PUL. As the device contains metal, artefact reduction protocols can be instituted to reduce the artefact generated from the device. Well known protocols include the MAVRIC SL sequence, which has been shown to reduce metal artefact on 3 T MRI.^23^ In our study, the artefact reduction protocol was used for the T2 phase and was shown to reduce the artefact by a mean of 2.3 mm but was found to also reduce image resolution and quality. T2 is generally utilised to assess the transitional zone. The multiphase phases of MRI used to assess the peripheral zone include DWI, ADC and DCE. Whilst DWI artefact was shown to be large (mean 28.17 mm), importantly, it was shown to only obscure the TZ of the prostate and did not affect the PZ for assessment. ADC mapping also was shown to reduce PUL artefact and also preserve the PZ. No phase used to assess the PZ was affected by PUL artefact, and, thus, no artefact reduction protocol is necessary for DWI, ADC and DCE.

For men with BPH, PUL has shown distinct advantages of a more rapid recovery than standard surgery and a unique freedom from iatrogenic sexual dysfunction, which are of particular importance to a significant portion of patients. MRI utilisation in prostate cancer surveillance and biopsy is now an established norm offering greater accuracy in cancer detection. It is of increasing importance to understand how best to offer both technologies so that patients can have continued access to these benefits. This study shows that the only zone of the prostate with compromised MR imaging is the posterior transition zone. It is encouraging that in our centre's database of prostate biopsies, no cancer diagnosis would have been missed due to this artefact. A potential clinical scenario of a completely normal mp‐MRI, with no lesions in the PZ or TZa, PUL artefact present in the TZp and raised PSA may arise. In this clinical scenario, if there is concern of missing TZp lesions suspicious for prostate cancer hidden by artefact, the recommendation would be to undertake targeted biopsies of this area. Importantly, the artefact that occurs from the device on MRI is not detected on ultrasound at the time of prostate biopsy, which allows surgeons to cognitively target these areas. For those patients being considered for a PUL, who are either being monitored for prostate cancer or who are on active surveillance, an MRI prior to device insertion should be considered. For those patients who have persistently elevated PSA readings who return with negative biopsies, repeating a biopsy with cognitive fusion of artefact zones on MRI if not done previously should also be contemplated.

The limitations of this study mainly centres upon there being no direct comparison to appreciate the effect of the artefact generated from the PUL in patients with known lesions identified on MRI pre‐ and post‐device insertion. In clinical practice, this will be difficult to achieve, as patients undergoing investigations for the diagnosis of prostate cancer are simultaneously unlikely to undergo PUL insertion at a similar point in time. Second, it is possible that in our cohort of patients who underwent a prostate biopsy, there may have been an under‐sampling of the posterior transition zone, particularly for those patients who had no suspicious lesions identified on MRI in this area. This is because generally the posterior transition zone is less likely to be sampled in a standard transperineal template biopsy with no suspicious lesions identified on MRI, mainly due to reduced incidence of prostate cancer occurring in this area and the risk of causing acute urinary retention post‐procedure. Third, in regards to mp‐MRI, given that there is artefact generated from the device, there is still potential whilst low and not demonstrated in our cohort of 10 patients that small lesions could still be obscured by the device, particularly seen in the DWI phase, which does play an important role in the classification of PIRADS 2 and 3 lesions identified initially on the T2 phase.^24^

## 5 CONCLUSIONS

This study has identified that MRI and PUL are compatible, and the device is unlikely to obscure a diagnosis of prostate cancer. In the rare circumstance where a lesion in the posterior transition zone is suspected, urologists should consider undertaking targeted prostate biopsies of this area.

## AUTHOR CONTRIBUTIONS

**Cameron Parkin:** Manuscript preparation, reference generation, collation of data. **Peter Chin:** Principal investigator, surgical proceduralist, manuscript preparation, data collection, trial co‐ordinator. **Rajeec Jyoti:** Radiologist consultant, data collection, database maintenance, MRI reporting, manuscript preparation.

## CONFLICT OF INTEREST STATEMENT

Neotract Inc/Teleflex funded the 10 patients who underwent magnetic resonance imaging prior to and following prostatic urethral lift insertion for this study. Dr. Cameron Parkin has no conflict of interests to declare. Dr. Rajeev Jyoti has no conflict of interests to declare. Associate Professor Peter Chin is a trainer and consultant for Teleflex.

## Data Availability

De‐identified data is available at request.
